# Adoption of high-sensitivity cardiac troponin for risk stratification of patients with suspected myocardial infarction: a multicentre cohort study

**DOI:** 10.1016/j.lanepe.2024.100960

**Published:** 2024-06-13

**Authors:** Michael McDermott, Dorien M. Kimenai, Atul Anand, Zen Huang, Andrew Houston, Sophie Williams, Felicity Evison, Suzy Gallier, Catalina Carenzo, Ben Glampson, Madina Hasan, Alexander Robertson, Thomas Phillips, Cai Davis, Elizabeth Sapey, Erik Mayer, Suzanne Mason, Matthew Stammers, Nicholas L. Mills, HDRUK Regional Linked Data Driven Evidence Network

**Affiliations:** a British Heart Foundation (BHF) Centre for Cardiovascular Science, University of Edinburgh, Edinburgh, UK; b Usher Institute, University of Edinburgh, Edinburgh, UK; c Bart's Health Life Science, Bart's Health NHS Trust, London, UK; d PIONEER Health Data Hub and NIHR Birmingham Biomedical Research Centre, Institute of Inflammation and Ageing, University of Birmingham, Birmingham, UK; e Imperial Clinical Analytics, Research & Evaluation (iCARE) Secure Data Environment, NIHR Imperial Biomedical Research Centre, St Mary's Hospital, London, UK; f CURE Group, Sheffield Centre for Health and Related Research, The University of Sheffield, Sheffield, UK; g Research Data Sciences Team, SETT Centre, University Hospital Southampton, Southampton, UK

**Keywords:** Healthcare data, Data linkage, High-sensitivity cardiac troponin, Healthcare outcomes, Myocardial infarction

## Abstract

**Background:**

Guidelines recommend high-sensitivity cardiac troponin to risk stratify patients with possible myocardial infarction and identify those eligible for discharge. Our aim was to evaluate adoption of this approach in practice and to determine whether effectiveness and safety varies by age, sex, ethnicity, or socioeconomic deprivation status.

**Methods:**

A multi-centre cohort study was conducted in 13 hospitals across the United Kingdom from November 1st, 2021, to October 31st, 2022. Routinely collected data including high-sensitivity cardiac troponin I or T measurements were linked to outcomes. The primary effectiveness and safety outcomes were the proportion discharged from the Emergency Department, and the proportion dead or with a subsequent myocardial infarction at 30 days, respectively. Patients were stratified using peak troponin concentration as low (<5 ng/L), intermediate (5 ng/L to sex-specific 99th percentile), or high-risk (>sex-specific 99th percentile).

**Findings:**

In total 137,881 patients (49% [67,709/137,881] female) were included of whom 60,707 (44%), 42,727 (31%), and 34,447 (25%) were stratified as low-, intermediate- and high-risk, respectively. Overall, 65.8% (39,918/60,707) of low-risk patients were discharged from the Emergency Department, but this varied from 26.8% [2200/8216] to 93.5% [918/982] by site. The safety outcome occurred in 0.5% (277/60,707) and 11.4% (3917/34,447) of patients classified as low- or high-risk, of whom 0.03% (18/60,707) and 1% (304/34,447) had a subsequent myocardial infarction at 30 days, respectively. A similar proportion of male and female patients were discharged (52% [36,838/70,759] *versus* 54% [36,113/67,109]), but discharge was more likely if patients were <70 years old (61% [58,533/95,227] *versus* 34% [14,428/42,654]), from areas of low socioeconomic deprivation (48% [6697/14,087] *versus* 43% [12,090/28,116]) or were black or asian compared to caucasian (62% [5458/8877] and 55% [10,026/18,231] *versus* 46% [35,138/75,820]).

**Interpretation:**

Despite high-sensitivity cardiac troponin correctly identifying half of all patients with possible myocardial infarction as being at low risk, only two-thirds of these patients were discharged. Substantial variation in the discharge of patients by age, ethnicity, socioeconomic deprivation, and site was observed identifying important opportunities to improve care.

**Funding:**

10.13039/501100000274UK Research and Innovation.

**Research in context**

**Evidence before this study**

We searched PubMed for reports, meta-analysis, randomised controlled trials and guidelines published in English between January 1, 2010, and December 31, 2023 with the search terms “high sensitivity cardiac troponin”, “cardiac troponin”, “myocardial infarction”, “acute coronary syndrome”, “myocardial injury”, “early rule out pathways”, “elderly patients”. “health care outcomes for ethnic groups”, and “social deprivation”. Data from observational studies, randomised trials and meta-analyses have shown the effectiveness and safety of early rule out pathways for myocardial infarction that use high-sensitivity cardiac troponin to risk stratify patients. However, most studies enrolled selected patients that may not be representative, and therefore it is unclear whether the performance of risk stratification with high-sensitivity cardiac troponin varies by age, sex, ethnicity, or social deprivation status. This uncertainty may have limited adoption in practice.

**Added value of this study**

We evaluated 137,881 consecutive patients attending Emergency Departments with possible myocardial infarction across 13 hospitals within the United Kingdom. Half of all patients with possible myocardial infarction were identified as low-risk by high-sensitivity cardiac troponin. These patients were very low risk, with just 1 in 1000 experiencing an index myocardial infarction and 1 in 3000 having a subsequent myocardial infarction at 30 days following discharge. Despite this only two-thirds of these patients were discharged with substantial variation by age, deprivation, ethnicity, and site.

**Implications of all the available evidence**

Our findings identify important opportunities to improve care for patients with possible myocardial infarction and prevent unnecessary hospital admission through the consistent application of risk stratification with high-sensitivity cardiac troponin in practice.

## Introduction

Chest pain is the most common reason for presentation to the Emergency Department worldwide, with more than 15 million attendances each year in Europe and the United States alone, accounting for 10% of hospital visits and 40% of admissions.^1^, ^2^, ^3^ Given the majority of patients do not have myocardial infarction, effective and safe care pathways that avoid unnecessary hospital admissions is an international healthcare priority.

High-sensitivity cardiac troponin assays with enhanced precision at very low concentrations have improved the risk stratification of patients with possible myocardial infarction.^4^, ^5^, ^6^ Multiple observational studies suggest early rule–out pathways using high-sensitivity cardiac troponin could facilitate discharge of the majority of patients from the Emergency Department.^7^, ^8^, ^9^, ^10^ The effectiveness and safety of this strategy has recently been demonstrated in randomised controlled trials,^11^, ^12^, ^13^ with single and serial-sample rule–out pathways now recommended by multiple international clinical guidelines.^14^, ^15^, ^16^, ^17^, ^18^

Implementation of early rule–out pathways using high-sensitivity cardiac troponin could save healthcare resources without compromising safe patient care. However, most studies have enrolled selected patients that may not be representative or have not been large enough to evaluate performance in key subgroups, and therefore it is unclear whether the effectiveness and safety of rule–out pathways varies by age, sex, ethnicity, or socioeconomic deprivation status. Whether this uncertainty has limited adoption in practice is unknown. Our aim was to use routine data from consecutive patients, and through federated analytics, understand variation in the adoption of high-sensitivity cardiac troponin for the risk stratification of patients with possible myocardial infarction across the United Kingdom, and to provide insight into the effectiveness and safety of this approach by key subgroups.

## Methods

### Study design and population

A multi-centre cohort study was conducted across 13 secondary or tertiary care hospitals in the United Kingdom using routinely collected linked healthcare data. All participating sites were part of the Health Data Research UK (HDRUK) Regional Linked Data Driven Evidence Network. Consecutive adult patients with possible myocardial infarction attending an Emergency Department were included during a 12-month period between 1 November 2021 and 31 October 2022. All patients who had one or more cardiac troponin measurement using a high-sensitivity assay within 24 h of presentation were included. If a patient attended more than once during the study period, only the first presentation was included.

The study was classified as a service evaluation with individual patient level data accessed and linked with approval of local Research Ethics Committees and Caldicott Guardians, following the Reporting of studies Conducted using Observational Routinely collected Data (RECORD) guidelines.

### Data collection

Data were collected by the HDRUK Regional Linked Data Driven Evidence Network using both the electronic patient record and regional or national datasets. In England, safety outcomes were obtained directly from the electronic patient record at each site, using the data that is submitted for national Hospital Episode Statistics. In Scotland, these outcomes were obtained from the national Scottish Morbidity Record. Individual patient-level data was deidentified and aggregated into pre-specified tables at site level. Tables comprising aggregate data from each site underwent disclosure review and minimisation to reduce the risk of disclosure by the local information governance team in accordance to the Working Group for Safe Data Access Professionals Handbook on Statistical Disclosure Control for Outputs.^19^

### Clinical characteristics

For each site, we collected clinical characteristics of each patient at baseline by linking laboratory data with primary and secondary care data from the electronic patient record (Supplementary Figure S1). Individual patient-level data were collected at each site for age, sex, ethnicity, social deprivation status, past medical history, smoking status and estimated glomerular filtration rate. We recorded the cardiac troponin assay used at each site and whether adjuvant risk scores were used within the care pathway. Adjuvant risk scores, such as the HEART score,^20^ can be used in chest pain pathways to support clinical decisions and aid in risk stratification.

### High-sensitivity cardiac troponin measurements

All sites used a high-sensitivity cardiac troponin assay to guide clinical care during the study period (Supplementary Table S1). Cardiac troponin I or T concentrations were measured by Abbott ARCHITECT_STAT_ high-sensitive troponin I assay (Abbott Laboratories) or Roche Elecsys high-sensitivity cardiac troponin T assay (Roche Diagnostics), respectively. The Abbott ARCHITECT_STAT_ high-sensitive troponin I assay has a limit of detection of 1.2 ng/L, an inter-assay coefficient of variation of less than 10% at 4.7 ng/L, and a 99th centile upper reference limit of 34 ng/L in men and 16 ng/L in women.^21^ This Roche Elecsys high-sensitivity cardiac troponin T assay has a limit of detection of 3 ng/L, an inter-assay coefficient of variation of less than 10% at 13 ng/L, and a 99th centile upper reference limit of 16 ng/L in men and 9 ng/L in women.^22^

Triage to discharge, further observation, or hospital admission is based on the risk (or probability) of whether the presentation is a consequence of myocardial infarction. The cardiac troponin concentration for the risk stratification of patients with possible myocardial infarction as low risk is <5 ng/L for both assays.^23^^,^^24^ Using serial measurements and including the maximal cardiac troponin concentration measured within 24 h of presentation, we classified patients into three groups according to guideline recommended thresholds: low-risk (<5 ng/L), intermediate-risk (5 ng/L to sex-specific 99th centile upper reference limit) and high-risk (>sex-specific 99th centile upper reference limit) of myocardial infarction or death within 30 days.^15^

### Effectiveness and safety outcomes

The primary effectiveness outcome was the proportion of patients discharged from the Emergency Department. The primary safety outcome was a composite of death from any cause during index admission or within 30 days of discharge, or subsequent myocardial infarction within 30 days of discharge. Patients were classified as having a myocardial infarction if their maximum cardiac troponin concentration was >sex specific 99th centile upper reference limit and they had a 10th revision of the International Classification of Diseases (ICD-10) code of I21 or I22. Secondary outcomes included the proportion of patients with an index myocardial infarction, subsequent myocardial infarction, any reattendance, death from any cause, cardiac death (ICD-10 diagnostic codes I05–I09, I20–I25, and I30–I5, limited to position 1 or 2 on the death record), and cardiovascular death (ICD-10 diagnostic codes I00–I99, limited to position 1 or 2 on the death record) within 30 days of discharge.

### Subgroups

For both the primary effectiveness and safety outcome we evaluated performance of high sensitivity cardiac troponin in pre-specified subgroups. We evaluated performance by age (less than 70 years of age or 70 years or greater), sex (female, male), ethnicity (white, asian, black or other) and socioeconomic deprivation status (group 1 [most deprived, quintile 1], group 2 [quintiles 2–4] or group 3 [least deprived, quintile 5]). Stratification by age was based on the average age of patients with possible myocardial infarction and evidence of myocardial injury from prior studies.^25^ Socioeconomic deprivation status was determined using the Scottish Index of Multiple Deprivation score or the Townsend Score in England, both are area based measures of socioeconomic deprivation.^26^^,^^27^ Patients were stratified by quintile into three groups as previously described.^28^

### Statistical analysis

Clinical characteristics and outcome variables are presented as absolute numbers (%) for the entire cohort and stratified by cardiac troponin groups (low-risk, intermediate-risk and high-risk). For the primary effectiveness outcome, we determined the proportion of patients discharged from Emergency Department across all sites and by individual site. For the primary safety outcome, we determined the negative predictive value of the rule-out threshold of <5 ng/L in all patients and by site. In a secondary analysis, we determined the negative predictive value of the rule-out threshold of <5 ng/L for both index and subsequent myocardial infarction within 30 days. We also evaluated outcomes by site with stratification according to the type of cardiac troponin assay, use of adjuvant risk scores and whether the site was a secondary or tertiary care centre.

A binomial likelihood with an equal-tailed Jeffreys prior was used to estimate the 95% confidence intervals around the negative predictive values.^29^ We evaluated the performance of high sensitivity cardiac troponin in pre-specified subgroups by age, sex, ethnicity and socioeconomic deprivation status. A Chi-square test was used to determine statistical difference. To allow for multiple testing, we applied the Bonferroni correction, and we therefore considered p < 0.005 (0.05/10) to provide evidence of association.

To further understand our main analysis, we conducted a *post hoc* and exploratory analyses in a nested substudy where we pooled individual patient-level data from three participating sites. We applied univariable and multivariable logistic regression modelling for the primary effectiveness and primary safety outcomes by cardiac troponin group and for each patient subgroup. The multivariable models were adjusted for age, sex, ethnicity, and socioeconomic deprivation. In this substudy, we also performed a sensitivity analysis to evaluate effectiveness and safety outcomes in patients presenting with chest pain.

All data were aggregated for analysis within a Secure Data Environment (DataLoch, Edinburgh, United Kingdom) with the analysis conducted using R (version 4.2.0; The R Foundation for Statistical Computing). The original R code for this study is available upon reasonable request.

### Role of the funding source

The funders of this study had no role in study design, data collection, data analysis, data interpretation, or writing of the report.

## Results

### Clinical characteristics of study population

The study population included 137,881 consecutive patients (48.7% females, 30.9% > 70 years old) who attended an Emergency Department and had cardiac troponin measured within 24 h of presentation between 1 November 2021 and 31 October 2022. Across all sites 60,707 (44.0%), 42,727 (31.0%), and 34,447 (25%) patients with possible myocardial infarction were stratified as low-, intermediate-, or high-risk, respectively. Patients who were classified as low-risk were more likely to be female, younger, have fewer cardiovascular risk factors, less co-morbidity and were less likely to have previously undergone coronary revascularisation compared to the high-risk group (Table 1, Supplementary Table S1).

**Table 1:** Baseline characteristics of patients with possible myocardial infarction stratified by high-sensitivity cardiac troponin into low- (<5 ng/L), intermediate- (5 ng/L to sex-specific 99th percentile), and high-risk (>sex-specific >99th percentile) groups.

Variable	All	Low-risk	Intermediate	High-risk
n	137,881	60,707	42,727	34,447
Women n (%)	67,109 (48.7%)	34,938 (57.6%)	15,603 (36.5%)	16,568 (48.1%)
**Age**				
18–39 years n (%)	28,001 (20.3%)	21,790 (35.9%)	4871 (11.4%)	1340 (3.9%)
40–49 years n (%)	19,197 (13.9%)	12,098 (19.9%)	5284 (12.4%)	1815 (5.3%)
50–59 years n (%)	24,798 (18%)	12,136 (20%)	8724 (20.4%)	3938 (11.4%)
60–69 years n (%)	23,231 (16.8%)	7884 (13%)	9142 (21.4%)	6205 (18%)
70–79 years n (%)	21,526 (15.6%)	4665 (7.7%)	8270 (19.4%)	8591 (24.9%)
80 years or above n (%)	21,128 (15.3%)	2134 (3.5%)	6436 (15.1%)	12,558 (36.5%)
**Ethnicity**				
White n (%)	75,820 (65.6%)	25,863 (57.1%)	26,930 (69.6%)	23,027 (72.7%)
Asian n (%)	18,231 (15.8%)	7988 (17.6%)	6239 (16.1%)	4004 (12.6%)
Black n (%)	8877 (7.7%)	3688 (8.1%)	2851 (7.4%)	2338 (7.4%)
Other n (%)	12,666 (11%)	7720 (17.1%)	2647 (6.8%)	2299 (7.3%)
**Deprivation**				
Group 1, most deprived n (%)	44,246 (39.4%)	17,636 (39.3%)	15,549 (40.5%)	11,061 (38%)
Group 2 n (%)	53,566 (47.7%)	22,021 (49.1%)	17,721 (46.2%)	13,824 (47.5%)
Group 3, least deprived n (%)	14,482 (12.9%)	5180 (11.6%)	5089 (13.3%)	4213 (14.5%)
**Past medical history**				
Hypertension n (%)	41,902 (33.1%)	10,037 (17.5%)	15,719 (40.3%)	16,146 (53.6%)
Diabetes mellitus n (%)	20,609 (16.3%)	5215 (9.1%)	7287 (18.7%)	8107 (26.9%)
Myocardial infarction n (%)	11,247 (8.9%)	1604 (2.8%)	4166 (10.7%)	5477 (18.2%)
Heart failure n (%)	7073 (5.6%)	427 (0.7%)	1939 (5%)	4707 (15.6%)
Stroke n (%)	4218 (3.3%)	743 (1.3%)	1614 (4.1%)	1861 (6.2%)
Previous PCI n (%)	4012 (3.7%)	828 (1.7%)	1503 (4.5%)	1681 (6.4%)
Previous CABG n (%)	796 (0.7%)	131 (0.3%)	307 (0.9%)	358 (1.4%)
**Smoking**				
Current n (%)	10,233 (25.9%)	3333 (31.4%)	3891 (25.6%)	3009 (21.9%)
Former n (%)	13,461 (34.1%)	2816 (26.6%)	5353 (35.2%)	5292 (38.6%)
Never n (%)	15,838 (40.1%)	4451 (42%)	5967 (39.2%)	5420 (39.5%)
**eGFR**				
Over 60 n (%)	95,243 (80.9%)	53,249 (95.8%)	26,927 (79.3%)	15,067 (53.5%)
31 to 60 n (%)	17,620 (15%)	2158 (3.9%)	6081 (17.9%)	9381 (33.3%)
0 to 30 n (%)	4857 (4.1%)	180 (0.3%)	969 (2.9%)	3708 (13.2%)
**Cardiac troponin testing**				
Single test n (%)	94,319 (68.4%)	52,982 (87.3%)	29,948 (70.1%)	11,389 (33.1%)
Serial testing (2 or more tests) n (%)	43,562 (31.6%)	7725 (12.7%)	12,779 (29.9%)	23,058 (66.9%)
**Data available**				
Ethnicity	115,594 (83.8%)	45,259 (74.6%)	38,667 (90.5%)	31,668 (91.9%)
Deprivation	112,294 (81.4%)	44,837 (73.9%)	38,359 (89.8%)	29,098 (84.5%)
Hypertension	126,528 (91.8%)	57,378 (94.5%)	39,048 (91.4%)	30,102 (87.4%)
Diabetes mellitus	126,528 (91.8%)	57,378 (94.5%)	39,048 (91.4%)	30,102 (87.4%)
Myocardial infarction	126,528 (91.8%)	57,378 (94.5%)	39,048 (91.4%)	30,102 (87.4%)
Heart Failure	126,528 (91.8%)	57,378 (94.5%)	39,048 (91.4%)	30,102 (87.4%)
Stroke	126,528 (91.8%)	57,378 (94.5%)	39,048 (91.4%)	30,102 (87.4%)
Percutaneous coronary intervention	107,483 (78%)	48,047 (79.1%)	33,346 (78%)	26,090 (75.7%)
Coronary artery bypass graft	107,483 (78%)	48,047 (79.1%)	33,346 (78%)	26,090 (75.7%)
Smoking	39,532 (28.7%)	10,600 (17.5%)	15,211 (35.6%)	13,721 (39.8%)
eGFR	117,720 (85.4%)	55,587 (91.6%)	33,977 (79.5%)	28,156 (81.7%)

PCI, percutaneous coronary intervention; CABG, coronary artery bypass grafting; eGFR, estimated glomerular filtration rate.

### Primary effectiveness outcome by site and risk group

**Fig. 1:**
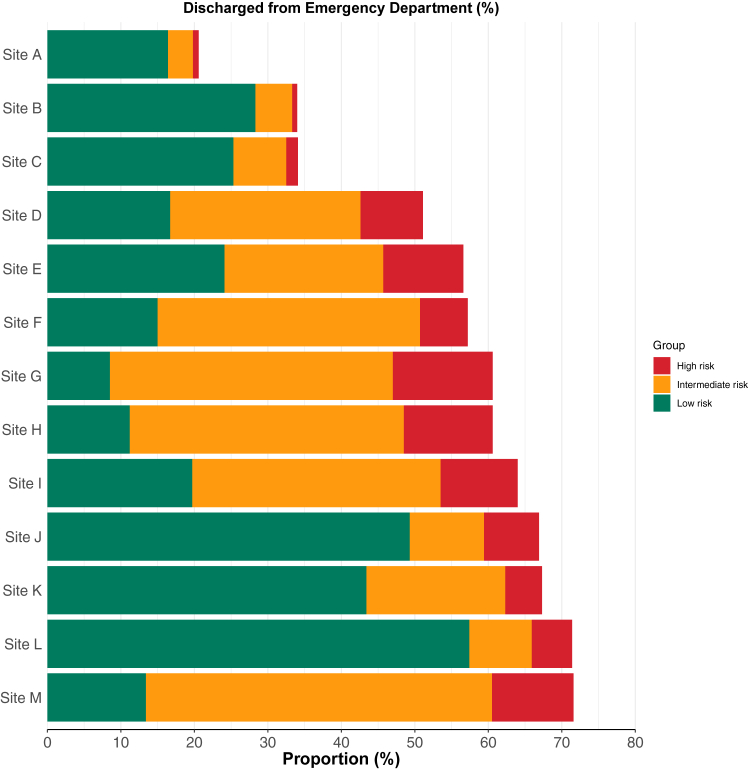
**The primary effectiveness outcome oftheproportion discharged from the Emergency Department stratified by high-sensitivity cardiac troponin concentration into low- (<5 ng/L), intermediate- (5 ng/L to sex-specific 99th percentile), and high risk (sex-specific >99th percentile) groups by hospital site**.

The primary effectiveness outcome of discharge from the Emergency Department occurred in 52.9% (72,961/137,881) of all patients in the study population (Table 2, Supplementary Table S2). The proportion of all patients discharged directly from the Emergency Department varied substantially across sites from 20.6% to 71.6% (p < 0.001) (Fig. 1, Supplementary Table S2). In total 60,707 (44.0%) patients were classified as low-risk with cardiac troponin concentrations <5 ng/L, however, only 65.8% (39,918/60,707) of these low-risk patients were discharged. When stratified by site, the proportion of low-risk patients discharged also varied substantially from 26.8% to 93.5% (p < 0.001) (Fig. 1, Supplementary Table S2).

**Table 2:** Effectiveness and safety outcomes for patients with possible myocardial infarction stratified by high-sensitivity cardiac troponin into low- (<5 ng/L), intermediate- (5 ng/L to sex-specific 99th percentile), and high-risk (>sex-specific >99th percentile) groups.

Variable	All	Low-risk	Intermediate	High-risk
n	137,881	60,707	42,727	34,447
**Primary effectiveness outcome**				
Discharged from Emergency Department n (%)	72,961 (52.9%)	39,918 (65.8%)	24,706 (57.8%)	8337 (24.2%)
**Primary safety outcome**				
All-cause death or myocardial infarction at 30 days n (%)	5126 (3.7%)	277 (0.5%)	932 (2.2%)	3917 (11.4%)
**Secondary outcomes**				
Index myocardial infarction n (%)	4919 (3.6%)	74 (0.1%)	179 (0.4%)	4666 (13.5%)
Subsequent myocardial infarction within 30 days n (%)	409 (0.4%)	18 (0.03%)	87 (0.2%)	304 (1%)
All-cause death during admission or within 30 days of discharge n (%)	3985 (2.9%)	230 (0.4%)	813 (1.9%)	2942 (8.5%)
Cardiac death within 30 days n (%)	247 (0.4%)	12 (0.02%)	81 (0.4%)	154 (1.2%)
Cardiovascular death within 30 days n (%)	340 (0.6%)	32 (0.1%)	110 (0.6%)	198 (1.6%)
All-cause death within 30 days of discharge n (%)	877 (0.9%)	60 (0.1%)	239 (0.7%)	578 (2.3%)
Any reattendance within 30 days n (%)	19,353 (14%)	6504 (10.7%)	6877 (16.1%)	5972 (17.3%)
Coronary angiography within 30 days n (%)	1826 (1.7%)	67 (0.1%)	155 (0.5%)	1604 (6.1%)
Coronary revascularisation within 30 days n (%)	1958 (1.8%)	172 (0.4%)	263 (0.8%)	1523 (5.8%)

Although the majority of patients who were classified as high-risk with cardiac troponin concentrations above the sex-specific 99th percentile were admitted to hospital, 24.2% (8337/34,447) were discharged from the Emergency Department. When stratified by site, the proportion of high-risk patients discharged also varied substantially from 4.9% to 34.8% (p < 0.001) (Fig. 1, Supplementary Table S2).

### Primary safety outcome by site and risk group

**Fig. 2:**
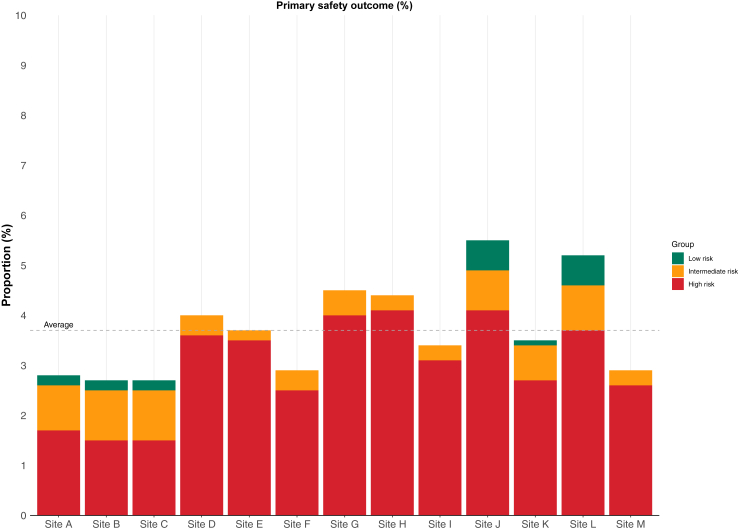
**The primary safety outcome of all cause death or myocardial infarction at 30 days stratified by high-sensitivity cardiac troponin concentration into low- (<5 ng/L), intermediate- (<5 ng/L to sex-specific 99th percentile), and high risk (sex-specific >99th percentile) groups by hospital site**.

**Fig. 3:**
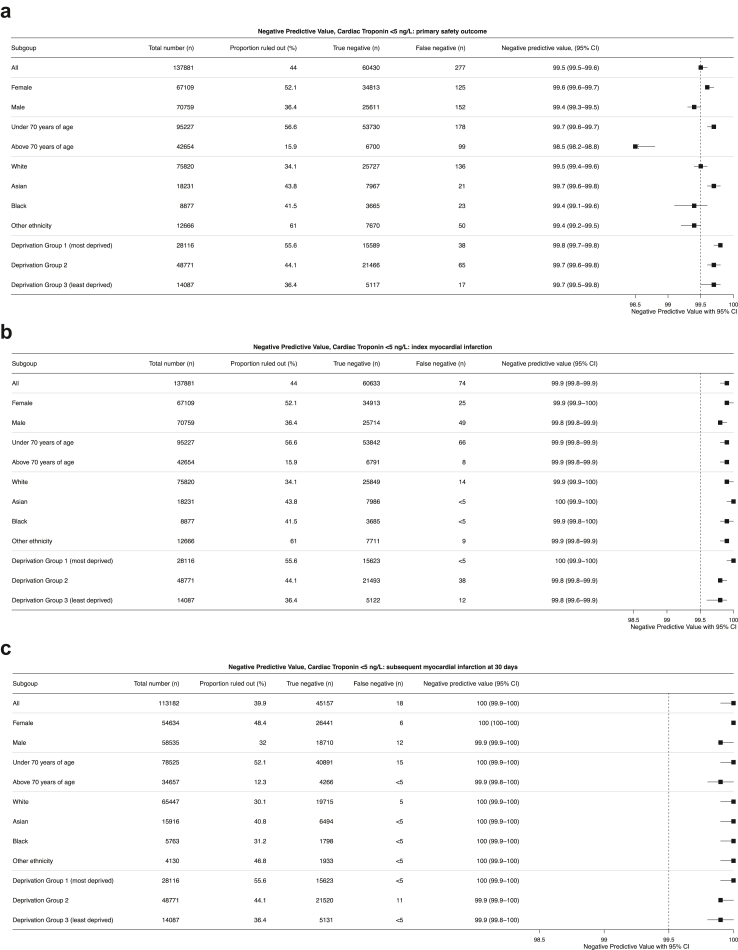
**Negative predictive value of high-sensitivity cardiac troponin I or T concentrations <5 ng/L for a) primary safety outcome (composite of all cause death or subsequent myocardial infarction) at 30 days from hospital discharge by subgroups, b)index myocardial infarction by subgroups and c)subsequent myocardial infarction within 30 days of hospital discharge**.

The primary safety outcome of death from any cause or subsequent myocardial infarction within 30 days of discharge occurred in 5126 (3.7%) patients and varied by site from 2.7% to 5.6% (p < 0.001) (Table 2, Fig. 2 and Supplementary Table S2). In the 60,707 patients classified as low risk, the primary safety outcome occurred in 277 (0.5%) patients. In those who were identified as intermediate- or high-risk the safety outcome occurred in 932 (2.2%) and 3917 (11.4%) patients, respectively (Table 2). The negative predictive value for the primary safety outcome using a cardiac troponin concentration <5 ng/L was 99.5% (99.5% confidence interval [CI] 99.5–99.6, Fig. 3 Panel A and Supplementary Figure S2).

### Secondary outcomes by risk group

The key secondary outcomes of index and subsequent myocardial infarction within 30 days of discharge, occurred in 4919 (3.6%) and 409 (0.4%) patients, respectively (Table 2). In patients classified as low-risk, just 74 (0.1%) and 18 (0.03%) went on to have a diagnosis of myocardial infarction during the index hospital admission or within 30 days following discharge, respectively. In patients classified as high-risk with cardiac troponin concentrations above the 99th percentile, 4666 (13.5%) and 304 (1.0%) went on to have a diagnosis of myocardial infarction during the index hospital admission or within 30 days following discharge, respectively. For patients stratified as low-risk the rate of all-cause death, cardiac death, cardiovascular death, reattendance, coronary angiography and revascularisation were all lower than patients classified as intermediate- or high-risk (Table 2, Supplementary Tables S3, S4 and S5).

### Primary effectiveness outcomes by subgroups

When stratified by sex, no clinical difference was observed in the primary effectiveness outcome between males and females (52.1% *versus* 53.8%) with the same proportion discharged from the Emergency Department (Supplementary Table S3). In contrast, a difference was observed across subgroups by socioeconomic deprivation status (group 1 = 43.0% [most deprived], group 2 = 46.8%, group 3 = 47.5% [least deprived] and in older adults (over 70 years of age), who were less likely to be discharged than younger adults (33.8% *versus* 61.5%) (p < 0.001 for all).

Patients of white ethnicity were less likely to be discharged from the Emergency Department (35,138 [46.3%]) compared to patients of black (5458 [61.5%]), asian (10,026 [55.0%]) and other ethnic groups (8603 [67.9%]) (p < 0.001 for all). These differences were more marked when stratified by risk with 59.3% (15,349/25,863), 57.2% (4572/7988), 72.1% (2659/3688), and 79.9% (6167/7720) of low-risk patients from white, asian, black and other ethnic groups discharged rather than admitted for further investigation (p < 0.001 for all). Furthermore, there was clinically relevant variation by ethnicity for high-risk patients where 22.6% (5210/23,027), 28.8% (1153/4004), 32.4% (758/2338), and 30.8% (709/2299) from white, asian, black and other ethnic groups, respectively, were discharged from hospital despite being stratified as high-risk.

### Primary safety outcomes by subgroups

Safety outcomes differed by age, sex, ethnicity, and socioeconomic deprivation status (Supplementary Table S3). For example, patients over 70 years were more likely to have the primary safety outcome compared to younger patients (8.3% [3527/42,654]) *versus* 1.7% [1599/95,227]. Similarly, men were more likely to have the primary safety outcome compared to women (4.2% [2998/70,772]) *versus* 3.2% [2128/67,109]; p < 0.001). Overall, the primary safety outcome occurred more frequently in patients of white ethnicity (4.5% [3439/75,820) relative to patients of black (3.2% [282/8877]), asian (2.6% [482/18,231]) and other (3.8% [478/12,666]) ethnic groups (p < 0.001 for all). These differences were more marked in the high-risk group with 11.5% (2653/23,027), 9.3% (373/4004), 9.5% (222/2338), and 15.4% (353/2299) from white, asian, black and other ethnic groups experiencing a death from any cause or subsequent myocardial infarction within 30 days of discharge (p < 0.001 for all).

The negative predictive value for the primary safety outcome was similar in all sub-groups except for older patients, where it was 98.5% (CI 98.2%–98.8%) compared to 99.7% (CI 99.6%–99.7%) in younger patients (Fig. 3, Panel A). However, the negative predictive value for both index myocardial infarction and subsequent myocardial infarction within 30 days of discharge was similar by age and was >99.5% in all subgroups (Fig. 3, Panel B and C).

### Exploratory analyses

Sites who used adjuvant risk scores had more variability in the primary effectiveness outcome (20.6%–67.4%) than sites that did not (Supplementary Table S2, Supplementary Figure S3). Whether a patient was assessed at a secondary or tertiary care site did not appear to contribute to this variation, which ranged from 33.9% to 71.6% in secondary care and 20.6% to 67.3% in tertiary care sites (Supplementary Table S2, Supplementary Figure S4). More variation was observed at sites using a cardiac troponin I assay (20.6%–71.4%) compared to those sites using a cardiac troponin T assay (51.1%–71.6%) (Supplementary Table S2 and Supplementary Figure S5). This was despite a higher proportion of patients being identified as low-risk at sites using cardiac troponin I compared to cardiac troponin T (59.0% *versus* 30.0%) (Supplementary Tables S1 and S2). Whilst the proportion of patients discharged was lower in sites using cardiac troponin I compared to sites using cardiac troponin T (49.2% *versus* 59.2%), more patients required serial (two or more) measurements to achieve this (40.2% *versus* 26.6%, respectively) (Supplementary Tables S1 and S2).

In a nested substudy where individual-patient level data was available from three sites, the primary effectiveness and primary safety outcome was compared in 17,934 patients stratified by cardiac troponin group with adjustment for age, sex, ethnicity, and socioeconomic deprivation. Findings were similar to our main analysis (Supplementary Tables S6 and S7). In those patients in whom data on the primary presenting symptom were available, chest pain was the most common symptom (86% [11,874/13,771]) (Supplementary Table S8). Patients with chest pain were less likely to be discharged (Supplementary Table S9 and Supplementary Figure S6) and were less likely to experience the primary safety outcome compared to the main analysis population where patients presented with a wider range of symptoms (Supplementary Table S10). The negative predictive value of a cardiac troponin concentration <5 ng/L for the primary safety outcome was greater than 99.5% in all subgroups, whether the primary presenting symptom was any symptom or restricted to those with chest pain (Supplementary Figures S7 and S8).

## Discussion

In 137,881 consecutive patients with possible myocardial infarction presenting to 13 hospitals over a 12-month period, we used data and federated analytics across the United Kingdom to understand variation in the adoption of high-sensitivity cardiac troponin and in care by subgroups. The following findings are relevant to practice. First, we observed substantial variation in the proportion of patients discharged from the Emergency Department across sites which varied from 20.6% to 71.6%. Second, almost half of all patients with possible myocardial infarction have cardiac troponin concentrations below guideline recommended low-risk thresholds. Despite this, one third of low-risk patients were admitted for further investigation. Third, these patients are very low risk, with just 1 in 1000 experiencing an index myocardial infarction and 1 in 3000 having a subsequent myocardial infarction at 30 days following discharge. Fourth, cardiac troponin identified low- and high-risk patients consistently when stratified by sex, ethnicity, and socioeconomic deprivation status. Although the risk of the primary safety outcome occurring, was higher in older persons with low cardiac troponin concentrations, this was due to higher rates of all cause death rather than myocardial infarction. Finally, we observed significant variation in the proportion of patients admitted to hospital for further investigation by ethnicity and socioeconomic deprivation, with higher rates of discharge in black, asian and other ethnic groups and in those from areas of lower deprivation, including in those stratified as high-risk with cardiac troponin concentrations above the 99th percentile.

For patients identified as low-risk with cardiac troponin I or T concentrations <5 ng/L the proportion discharged varied from 26.8% to 93.5%. Whilst some low-risk patients may have required hospital admission for the investigation of other presenting symptoms, this alone could not explain the marked variation in care observed. We explored site level factors that could account for this variation. The proportion of patients discharged was similar whether sites provided secondary or tertiary care. However, the type of assay used influenced the proportion of patients identified as low-risk and the number of serial measurements required to enable discharge from the Emergency Department. There was also some evidence that the use of adjuvant risk scores was associated with lower rates of discharge. However, these site level factors are not unrelated and a larger study with more sites coupled with a qualitative assessment is needed to gain a more complete understanding of the factors that influence variation in care in the United Kingdom and across different healthcare systems. Given patients with cardiac troponin concentrations <5 ng/L were consistently at low risk of major adverse cardiovascular events at 30 days, across all sites and all subgroups, the application of risk stratification thresholds in practice represents a potentially important opportunity to improve care and reduce unnecessary healthcare resource use and hospital admission.

Another surprising and potentially important observation was that although patients with cardiac troponin concentrations above the sex-specific 99th percentile were more likely to be admitted to hospital for further investigation, almost 1 in 4 were discharged home. Again, this varied substantially by site with between 4.9% and 34.8% of high-risk patients discharged. Interestingly this may reflect the low specificity of cardiac troponin where only 13.5% of patients with values above the sex-specific 99th percentile received an index diagnosis of myocardial infarction. Clinicians appear to feel that hospital admission in patients with likely non-ischemic causes of acute and chronic myocardial injury may not be justified. This is despite those with elevated cardiac troponin being high-risk where 1 in 9 patients were dead or had a subsequent myocardial infarction at 30 days. Our findings are consistent with prior studies from multiple countries demonstrating that patients with myocardial injury have poor outcomes,^30^^,^^31^ and further highlight the need for research to develop evidence-based care pathways for patients with likely non-ischemic myocardial injury.^32^^,^^33^ This may be particularly relevant after a large randomised controlled trial demonstrated that implementation of high-sensitivity cardiac troponin was associated with longer stays and improved long-term outcomes in those patients identified with non-ischemic myocardial injury.^32^^,^^33^

Few studies have evaluated the impact of risk stratification using high-sensitivity cardiac troponin on care and outcomes of patients with possible myocardial infarction stratified by ethnicity.^34^ We observed that patients of black, asian or other ethnic groups were more likely to be discharged home from the Emergency Department compared to white patients. Furthermore, high-risk patients of black or other ethnicity with elevated cardiac troponin concentrations, were less likely to be admitted for further investigation or to undergo angiography or revascularisation. An important caveat to this observation is that the overall rate of death or myocardial infarction was lower in black and other ethnic groups and similar between patients of asian and white ethnicity. In previous studies better outcomes in patients from asian and black ethnicities after acute hospital admission have been observed.^35^ This may reflect a lower burden of cardiovascular disease or different thresholds to seek medical care within these patient groups. There was also significant variation in ethnicity across regions that may have contributed to variation in care. Furthermore, we observed that patients from areas with more socioeconomic deprivation were more likely to be admitted to hospital than those from areas with less deprivation. Patients from more deprived areas have been shown to have worse outcomes across all age ranges and higher admission rates to hospital, which may reflect a greater burden of co-morbidity.^36^^,^^37^ Further research is needed to understand the patient, societal and system factors that determine whether a patient's ethnicity and socioeconomic deprivation status directly influences the decision to discharge or admit to hospital for further investigation.

Our study has several strengths. By using routine healthcare data and electronic patient record systems, we were able to identify all consecutive patients presenting to Emergency Departments with possible myocardial infarction. As such, our study population was representative of current practice in the United Kingdom and was inclusive. All groups were well represented with half of the study population being female, broad representation from different ethnic groups, older adults, and from patients across the socioeconomic deprivation spectrum that are often underrepresented in clinical studies. However, there are also a number of important limitations. First, although we included a mix of secondary and tertiary care hospitals with reasonable geographical coverage, this was a convenience sample of sites participating in the HDRUK Regional Linked Data Driven Evidence Network. Whether our finding are generalisable to other parts of the United Kingdom or to other healthcare settings is uncertain. Second, as our inclusion criteria consisted of all patients who had cardiac troponin measured within 24 h of presentation, it is likely that we have included some patients who were not being assessed for possible myocardial infarction. However, the findings from our sensitivity analysis restricted to patients with chest pain were reassuring and consistent with the main analysis. Third, although individual patient-level data was linked at each site, aggregated data was pooled for the main analysis, and we were only able to undertake multivariate analyses using data from 3 of the 13 participating sites. As the characteristics of patients from different ethnic groups and areas of socioeconomic deprivation varied across sites, further studies to understand variation in care and outcomes using individual patient-level data across a larger number of sites are warranted. Fourth, although data was complete for cardiac troponin and our primary effectiveness and safety outcomes, data on cardiovascular risk factors, such as smoking was not consistently available across all sites. Finally, the diagnosis of myocardial infarction was based upon diagnostic codes and troponin concentrations above the sex-specific 99th centile and therefore some misclassification is likely. Unlike our prior work the diagnosis of myocardial infarction was not adjudicated,^31^ therefore we are not able to report whether there was variation by site or subgroup in the proportion of patients with a diagnosis of type 2 myocardial infarction or acute non-ischemic myocardial injury.^15^

In conclusion, high sensitivity cardiac troponin is a powerful tool to evaluate risk in patients with possible myocardial infarction. Half of all patients were identified as low-risk and could potentially be discharged safely from the Emergency Department. However, only two-thirds of these low-risk patients were discharged in practice with substantial variation by age, ethnicity, deprivation, and site. The use of high-sensitivity cardiac troponin for risk stratification in patients with possible myocardial infarction represents an important opportunity to improve care and reduce unnecessary hospital admissions.

## Contributors

MM, DK and NLM conceived the study and its design. The HDR UK Regional Linked Data Consortium acquired the data. MM and DK performed the analysis. MM, DK and NLM interpreted the data and drafted the manuscript. All authors revised the manuscript critically for important intellectual content. All authors provided their final approval of the version to be published. All authors are accountable for the work.

## Data sharing statement

The study used routine electronic health care data sources that linked, de-identified and aggregated individual patient level data that is held in a Secure Data Environment by DataLoch (https://dataloch.org/). The study data and analysis code can be accessed by individuals who have undertaken the necessary governance training on application to DataLoch and the HDRUK Regional Linked Data Driven Evidence Network.

## Declaration of interests

NLM has received honoraria or consultancy from Abbott Diagnostics, Roche Diagnostics, Siemens Healthineers, LumiraDx, and Pyros Laboratories. All other authors have no interests to declare.

## Appendix A Supplementary data

Adoption_supplement
