# Supplementary material for: Adoption of high-sensitivity cardiac troponin for risk stratification of patients with suspected myocardial infarction: a multicentre cohort study
Source: Lancet Reg Health Eur. 2024 Jun 13;43:100960. doi: 10.1016/j.lanepe.2024.100960 (PMC11227019; doi:10.1016/j.lanepe.2024.100960)
Supplement: Adoption_supplement [file mmc1.docx]

SUPPLEMENTARY APPENDIX

**Adoption of high-sensitivity cardiac troponin for risk stratification of patients with suspected myocardial infarction: a multicentre cohort study**

Michael McDermott, M.D.,^1*^ Dorien M Kimenai, Ph.D.,^1*^ Atul Anand, M.D.,^1^

Zen Huang, Ph.D.,^2^ Andrew Houston^3^, Sophie Williams, Ph.D.,^3^ Felicity Evison,^4^ Suzy Gallier,^4^ Catalina Carenzo,^5^ Ben Glampson,^5^ Madina Hasan, PhD,^6^ Alexander Robertson M.D.,^6^ Thomas Phillips,^7^ Cai Davis,^7^ Erik Mayer, M.D.,^5^ Elizabeth Sapey, M.D.,^4^ Suzanne Mason M.D.,^6^ Matthew Stammers, M.D.,^7^ Nicholas L. Mills, M.D.^1, 2^

*on behalf of the HDRUK Regional Linked Data Driven Evidence Network*

^1^ British Heart Foundation (BHF) Centre for Cardiovascular Science, University of Edinburgh, Edinburgh, UK.

^2^ Usher Institute, University of Edinburgh, Edinburgh, UK.

^3^ Bart's Health Life Science, Bart’s Health NHS Trust, London, UK

^4^ PIONEER Health Data Hub and NIHR Birmingham Biomedical Research Centre, Institute of Inflammation and Ageing, University of Birmingham, Birmingham, UK

^5^ Imperial Clinical Analytics, Research & Evaluation (iCARE) Secure Data Environment, NIHR Imperial Biomedical Research Centre, St Mary’s Hospital, London, UK.

^6^ CURE Group, Sheffield Centre for Health and Related Research, The University of Sheffield, Sheffield, UK.

^7^ Research Data Sciences Team, SETT Centre, University Hospital Southampton, Southampton, UK

* Contributed equally

**Corresponding Author:**

Professor Nicholas L Mills

BHF/University Centre for Cardiovascular Science

The University of Edinburgh

Edinburgh EH16 4SA

United Kingdom

Telephone: 0044 131 242 6515

E-mail: [nick.mills@ed.ac.uk](mailto:nick.mills@ed.ac.uk)

**Figures and Tables**

**Supplementary Table 1.** Baseline characteristics of patients with possible myocardial infarction by hospital site.

**Supplementary Table 2.** Effectiveness and safety outcomes by hospital site for patients with possible myocardial infarction stratified by high-sensitivity cardiac troponin into low- (<5 ng/l), intermediate- (5 ng/L to sex-specific 99^th^ percentile), and high-risk (>sex-specific >99^th^ percentile) groups.

**Supplementary Table 3.** Effectiveness and safety outcomes by subgroup for patients with possible myocardial infarction stratified by high-sensitivity cardiac troponin into low- (<5 ng/l), intermediate- (5 ng/L to sex-specific 99^th^ percentile), and high-risk (>sex-specific >99^th^ percentile) groups.

**Supplementary Table 4.** Management outcomes by hospital site for patients with possible myocardial infarction who were stratified by high-sensitivity troponin as high-risk (>sex-specific >99^th^ percentile).

**Supplementary Table 5.** Management outcomes by subgroup for patients with possible myocardial infarction who were stratified by high-sensitivity troponin as high-risk (>sex-specific >99^th^ percentile).

**Supplementary Table 6.** Logistic regression modelling, within our nested study (n=17,934), demonstrating the relationship between risk groups and the primary effectiveness and safety outcomes adjusted for age, sex, ethnicity, and socioeconomic deprivation.

**Supplementary Table 7.** Logistic regression modelling, within out nested study (n=17,934), demonstrating the relationship between subgroups and the primary effectiveness and safety outcomes adjusted for age, sex, ethnicity, and socioeconomic.

**Supplementary Table 8.** Presenting complaint leading to measurement of cardiac troponin across hospital sites D, F and I (n=17,934).

**Supplementary Table 9.** Primary effectiveness outcome for all patients presenting to hospital and for patients presenting to hospital with symptom recorded Sites D, F and I (n=13,771).

**Supplementary Table 10.** Primary safety outcome for all patients presenting to hospital and for patients presenting to hospital with symptom recorded at Sites 1, 2 and 3 (n=13,771).

**Supplementary Figure 1.** Overview of methodology using laboratory data (high sensitivity cardiac troponin) to identify patients who presented with possible myocardial infarction to allow characterisation of their demographics, risk factors and outcomes of their assessment in the Emergency Department.

**Supplementary Figure 2.** Negative predictive value of high-sensitivity cardiac troponin I or T concentrations <5 ng/L for the primary safety outcome (composite of all cause death or subsequent myocardial infarction at 30 days post hospital discharge) by hospital site, separated by high sensitivity cardiac troponin assay.

**Supplementary Figure 3.** Proportion of patients discharged from the emergency department stratified by high-sensitivity cardiac troponin concentration into low- (<5 ng/l), intermediate- (5 ng/L to sex-specific 99^th^ percentile), and high-risk (>sex-specific >99^th^ percentile) groups by hospital site and use of adjuvant risk score (HEART score) or not.

**Supplementary Figure 4.** Proportion of patients discharged from the emergency department stratified by high-sensitivity cardiac troponin concentration into low- (<5 ng/l), intermediate- (5 ng/L to sex-specific 99^th^ percentile), and high-risk (>sex-specific >99^th^ percentile) groups by hospital site and whether a site is a secondary or tertiary centre.

**Supplementary Figure 5.** Proportion of patients discharged from hospital (A) and admitted to hospital (B) stratified by high-sensitivity cardiac troponin concentration into low- (<5 ng/l), intermediate- (5 ng/L to sex-specific 99^th^ percentile), and high-risk (>sex-specific >99^th^ percentile) groups by hospital site and cardiac troponin assay.

**Supplementary Figure 6.** Proportion of patients discharged from hospital stratified by high-sensitivity cardiac troponin concentration into low- (<5 ng/l), intermediate- (5 ng/L to sex-specific 99^th^ percentile), and high-risk (>sex-specific >99^th^ percentile) groups by hospital site with any symptom being the presenting complaint (A) and with chest pain (B).

**Supplementary Figure 7.** Negative predictive value of high-sensitivity cardiac troponin I or T concentrations <5 ng/L for the primary safety outcome (composite of all cause death or subsequent myocardial infarction at 30 days post hospital discharge) for patients presenting with any symptom (n=13,771).

**Supplementary Figure 8.** Negative predictive value of high-sensitivity cardiac troponin I or T concentrations <5 ng/L for the primary safety outcome (composite of all cause death or subsequent myocardial infarction at 30 days post hospital discharge) for patients presenting with chest pain (n=11,874).

| **Variable** | **All** | **Site A** | **Site B** | **Site C** | **Site D** | **Site E** | **Site F** | **Site G** | **Site H** | **Site I** | **Site J** | **Site K** | **Site L** | **Site M** |
| --- | --- | --- | --- | --- | --- | --- | --- | --- | --- | --- | --- | --- | --- | --- |
| n | 137,881 | 13,444 | 17,812 | 11,993 | 10281 | 11,353 | 4,452 | 6,848 | 7,901 | 3,201 | 11,490 | 19,045 | 13,209 | 6,852 |
| Women n  (%) | 67,109 (48·7%) | 7,054 (52·5%) | 9,247 (51·9%) | 6,270 (52·3%) | 4710 (45·8%) | 5,513 (48·6%) | 2,088 (46·9%) | 2,567 (37·5%) | 3,294 (41·7%) | 1,748 (54·6%) | 5,741 (50%) | 9,412 (49·4%) | 6,734 (51%) | 2,731 (39·9%) |
| **Age** |  |  |  |  |  |  |  |  |  |  |  |  |  |  |
| 18 to 39 years n (%) | 28,001 (20·3%) | 2,883 (21·4%) | 4,582 (25·7%) | 2,393 (20%) | 1,775 (17·3%) | 1,456 (12·8%) | 690 (15·5%) | 985 (14·4%) | 953 (12·1%) | 618 (19·3%) | 2,141 (18·6%) | 4,984 (26·2%) | 3,411 (25·8%) | 1,130 (16·5%) |
| 40 to 49 years n (%) | 19,197 (13·9%) | 2,108 (15·7%) | 2,548 (14·3%) | 1,558 (13%) | 1,420 (13·8%) | 1,640 (14·4%) | 676 (15·2%) | 1,050 (15·3%) | 936 (11·8%) | 417 (13%) | 1,459 (12·7%) | 2,434 (12·8%) | 1,702 (12·9%) | 1,249 (18·2%) |
| 50 to 59 years n (%) | 24,798 (18%) | 2,366 (17·6%) | 2,838 (15·9%) | 2,045 (17·1%) | 1,996 (19·4%) | 2,315 (20·4%) | 950 (21·3%) | 1,458 (21·3%) | 1,498 (19%) | 509 (15·9%) | 1,951 (17%) | 3,018 (15·8%) | 2,209 (16·7%) | 1,645 (24%) |
| 60 to 69 years n (%) | 23,231 (16·8%) | 2,110 (15·7%) | 2,639 (14·8%) | 1,852 (15·4%) | 1,898 (18·5%) | 2,051 (18·1%) | 885 (19·9%) | 1,471 (21·5%) | 1,633 (20·7%) | 556 (17·4%) | 1,921 (16·7%) | 2,964 (15·6%) | 1,908 (14·4%) | 1,343 (19·6%) |
| 70 to 79 years n (%) | 21,526 (15·6%) | 1,976 (14·7%) | 2,566 (14·4%) | 2,004 (16·7%) | 1,722 (16·7%) | 1,931 (17%) | 730 (16·4%) | 944 (13·8%) | 1,386 (17·5%) | 566 (17·7%) | 1,917 (16·7%) | 2,965 (15·6%) | 1,995 (15·1%) | 824 (12%) |
| 80 years or above n (%) | 21,128 (15·3%) | 2,001 (14·9%) | 2,639 (14·8%) | 2,141 (17·9%) | 1,470 (14·3%) | 1,960 (17·3%) | 521 (11·7%) | 940 (13·7%) | 1,495 (18·9%) | 535 (16·7%) | 2,101 (18·3%) | 2,680 (14·1%) | 1,984 (15%) | 661 (9·6%) |
| **Ethnicity*** |  |  |  |  |  |  |  |  |  |  |  |  |  |  |
| White n (%) | 82,352 (66·8%) | 6,277 (60·3%) | 10,479 (67·6%) | 8,361 (84·8%) | 8,284 (92·1%) | 8,635 (85·9%) | 3,989 (96·2%) | 2,458 (37·2%) | 4,112 (54·6%) | 2,555 (91·2%) | 3,838 (34·1%) | 14,827 (90%) | 6,535 (49·9%) | 2,002 (30·8%) |
| Asian n (%) | 18,849 (15·3%) | 3,180 (30·5%) | 3,213 (20·7%) | 756 (7·7%) | 392 (4·4%) | 304 (3%) | 93 (2·2%) | 2,749 (41·6%) | 1,868 (24·8%) | 138 (4·9%) | 946 (8·4%) | 913 (5·5%) | 1,369 (10·5%) | 2,928 (45·1%) |
| Black n (%) | 9,070 (7·4%) | 478 (4·6%) | 935 (6%) | 399 (4%) | 122 (1·4%) | 463 (4·6%) | 29 (0·7%) | 801 (12·1%) | 1,186 (15·8%) | 42 (1·5%) | 1,465 (13%) | 318 (1·9%) | 1,649 (12·6%) | 1,183 (18·2%) |
| Other n (%) | 12,935 (10·5%) | 476 (4·6%) | 865 (5·6%) | 343 (3·5%) | 197 (2·2%) | 655 (6·5%) | 37 (0·9%) | 601 (9·1%) | 359 (4·8%) | 66 (2·4%) | 5,005 (44·5%) | 416 (2·5%) | 3,531 (27%) | 384 (5·9%) |
| **Deprivation*** |  |  |  |  |  |  |  |  |  |  |  |  |  |  |
| Group 1, most deprived n (%) | 50,241 (37·3%) | 7,136 (53·2%) | 7,071 (39·9%) | 3,145 (26·3%) | 1,684 (16·7%) | 4,380 (38·9%) | 772 (17·5%) | 5,796 (86·4%) | 3,958 (50·4%) | 492 (15·6%) | 3,390 (33·2%) | 3,436 (18·1%) | 2,605 (21·4%) | 6,376 (94·3%) |
| Group 2  n (%) | 68,730 (51%) | 5,200 (38·8%) | 9,557 (53·9%) | 6,216 (52·1%) | 5,932 (58·8%) | 5,364 (47·6%) | 3,013 (68·4%) | 813 (12·1%) | 3,628 (46·2%) | 1,658 (52·4%) | 6,333 (62·1%) | 11,831 (62·3%) | 8,831 (72·6%) | 354 (5·2%) |
| Group 3, least deprived n (%) | 15,689 (11·7%) | 1,068 (8%) | 1,090 (6·2%) | 2,578 (21·6%) | 2,481 (24·6%) | 1,528 (13·6%) | 617 (14%) | 102 (1·5%) | 265 (3·4%) | 1,013 (32%) | 482 (4·7%) | 3,712 (19·6%) | 725 (6%) | 28 (0·4%) |
| **Past Medical History*** |  |  |  |  |  |  |  |  |  |  |  |  |  |  |
| Hypertension n (%) | 41,902 (33·1%) | 6,123 (45·5%) | 7022 (39·4%) | 4,930 (41·1%) | 2,762 (26·9%) | # | 1,199 (26·9%) | 2,783 (40·6%) | 3,128 (39·6%) | 859 (26·8%) | 1,730 (15·1%) | 7,228 (38%) | 1,716 (13%) | 2,422 (35·3%) |
| Diabetes mellitus n (%) | 20,609 (16·3%) | 3,229 (24%) | 3359 (18·9%) | 2,015 (16·8%) | 1,279 (12·4%) | # | 629 (14·1%) | 1,743 (25·5%) | 1,474 (18·7%) | 386 (12·1%) | 1,039 (9%) | 3,117 (16·4%) | 892 (6·8%) | 1,447 (21·1%) |
| Myocardial infarction n (%) | 11,247 (8·9%) | 1,083 (8·1%) | 1412 (7·9%) | 807 (6·7%) | 1,126 (11%) | # | 503 (11·3%) | 846 (12·4%) | 894 (11·3%) | 240 (7·5%) | 189 (1·6%) | 3,279 (17·2%) | 184 (1·4%) | 684 (10%) |
| Heart Failure n (%) | 7,073 (5·6%) | 9 (0·1%) | 19 (0·1%) | 5 (0%) | 625 (6·1%) | # | 259 (5·8%) | 822 (12%) | 883 (11·2%) | 215 (6·7%) | 217 (1·9%) | 3,281 (17·2%) | 145 (1·1%) | 593 (8·7%) |
| Stroke n (%) | 4,218 (3·3%) | 260 (1·9%) | 465 (2·6%) | 195 (1·6%) | 117 (1·1%) | # | 58 (1·3%) | 245 (3·6%) | 265 (3·4%) | 43 (1·3%) | 212 (1·8%) | 1,960 (10·3%) | 222 (1·7%) | 176 (2·6%) |
| Previous PCI n (%) | 4,012 (3·7%) | 1,030 (7·7%) | 915 (5·1%) | 575 (4·8%) | 48 (0·5%) | # | <15 (*%) | 462 (6·7%) | 491 (6·2%) | <5 (*%) | 35 (0·3%) | # | 23 (0·2%) | 415 (6·1%) |
| Previous CABG n (%) | 796 (0·7%) | 39 (0·3%) | 154 (0·9%) | 46 (0·4%) | 72 (0·7%) | # | 41 (0·9%) | 123 (1·8%) | 113 (1·4%) | 21 (0·7%) | 51 (0·4%) | # | 56 (0·4%) | 80 (1·2%) |
| **Smoking status*** |  |  |  |  |  |  |  |  |  |  |  |  |  |  |
| Current n (%) | 10,233 (25·9%) | 252 (26·3%) | 1,010 (28·4%) | 174 (23·9%) | 1,926 (23·8%) | # | 890 (24·9%) | 581 (26·4%) | 480 (22·7%) | 585 (22·4%) | 860 (88·8%) | 2,080 (18%) | 903 (88·1%) | 492 (22·8%) |
| Former n (%) | 13,461 (34·1%) | 248 (25·9%) | 1,153 (32·5%) | 191 (26·3%) | 3,433 (42·4%) | # | 1,641 (45·8%) | 445 (20·2%) | 370 (17·5%) | 1,106 (42·4%) | 66 (6·8%) | 4,269 (37%) | 90 (8·8%) | 449 (20·8%) |
| Never n (%) | 15,838 (40·1%) | 459 (47·9%) | 1,389 (39·1%) | 362 (49·8%) | 2,739 (33·8%) | # | 1,049 (29·3%) | 1,177 (53·4%) | 1,261 (59·7%) | 919 (35·2%) | 42 (4·3%) | 5,189 (45%) | 32 (3·1%) | 1,220 (56·5%) |
| **eGFR*** |  |  |  |  |  |  |  |  |  |  |  |  |  |  |
| Over 60 n (%) | 95,243 (80·9%) | 11,251 (84·1%) | 14,814 (83·5%) | 9,928 (83%) | 8,435 (82·7%) | # | 3,605 (81·6%) | 2,736 (60·5%) | 3,596 (64·2%) | 2,480 (80·8%) | 9,409 (82·5%) | 15,801 (83·3%) | 11,118 (84·9%) | 2,070 (61·9%) |
| 31 to 60 n (%) | 17,620 (15%) | 1,616 (12·1%) | 2,244 (12·6%) | 1,667 (13·9%) | 1,537 (15·1%) | # | 683 (15·5%) | 1278 (28·3%) | 1,602 (28·6%) | 495 (16·1%) | 1,473 (12·9%) | 2,545 (13·4%) | 1,435 (11%) | 1,045 (31·2%) |
| 0 to 30 n (%) | 4,857 (4·1%) | 518 (3·9%) | 686 (3·9%) | 368 (3·1%) | 233 (2·3%) | # | 128 (2·9%) | 507 (11·2%) | 402 (7·2%) | 94 (3·1%) | 526 (4·6%) | 624 (3·3%) | 542 (4·1%) | 229 (6·8%) |
| **Number of Cardiac Troponin tests** |  |  |  |  |  |  |  |  |  |  |  |  |  |  |
| Single test n (%) | 94319 (68·4%) | 10161 (75·6%) | 12039 (67·6%) | 9046 (75·4%) | 4749 (46·2%) | 7431 (65·5%) | 1779 (40%) | 4449 (65%) | 5398 (68·3%) | 2040 (63·7%) | 8693 (75·7%) | 13154 (69·1%) | 10786 (81·7%) | 4594 (67%) |
| Serial testing (2 or more tests) n (%) | 43562 (31·6%) | 3283 (24·4%) | 5773 (32·4%) | 2947 (24·6%) | 5532 (53·8%) | 3922 (34·5%) | 2673 (60%) | 2399 (35%) | 2503 (31·7%) | 1161 (36·3%) | 2797 (24·3%) | 5891 (30·9%) | 2423 (18·3%) | 2258 (33%) |
| Troponin assays (I or T) |  | I | I | I | T | T | T | T | T | T | I | I | I | T |
| Adjuvant risk score (yes or no) |  | Yes (HEART) | Yes (HEART) | Yes (HEART) | No | No | No | No | No | No | No | Yes (HEART) | No | No |

*PCI = Percutaneous Coronary intervention*

*CABG = Coronary artery bypass graft*

*# Data unavailable*

**Data availability n (%): ethnicity 123,206 (89·4%), deprivation 134,660 (97·7%), past medical history126,528 (91·8%), PCI 107,483 (78%), CABG 107,483 (78%), smoking 39,532 (28·1%) and eGFR 117, 720 (85·4%)*

**Supplementary Table 1. Baseline characteristics of patients with possible myocardial infarction by hospital site.**

| **Outcome** | **Risk Stratification** | **All** | **Site A** | **Site B** | **Site C** | **Site D** | **Site E** | **Site F** | **Site G** | **Site H** | **Site I** | **Site J** | **Site K** | **Site L** | **Site M** |
| --- | --- | --- | --- | --- | --- | --- | --- | --- | --- | --- | --- | --- | --- | --- | --- |
| **Primary effectiveness outcome n (%)** | All | 72,961 (52·9%) | 2,764 (20·6%) | 6,045 (33·9%) | 4,089 (34·1%) | 5,252 (51·1%) | 6,421 (56·6%) | 2,551 (57·3%) | 4,150 (60·6%) | 4,789 (60·6%) | 2,047 (63·9%) | 7,696 (67%) | 12,820 (67·3%) | 9,429 (71·4%) | 4,908 (71·6%) |
|  | Low | 39,918 (65·8%) | 2,200 (26·8%) | 5,033 (45·8%) | 3,037 (42%) | 1,719 (87·6%) | 2,731 (82%) | 670 (80%) | 585 (89·9%) | 882 (92·5%) | 630 (92·8%) | 5,669 (83·3%) | 8,263 (88·6%) | 7,581 (86·8%) | 918 (93·5%) |
|  | Intermediate | 24,706 (57·8%) | 460 (14·4%) | 889 (20·7%) | 863 (27·5%) | 2,664 (59·4%) | 2,455 (66·7%) | 1,591 (73·8%) | 2,635 (79·9%) | 2,950 (79·4%) | 1,081 (79·8%) | 1,162 (57·3%) | 3,601 (63·2%) | 1,127 (56·6%) | 3,228 (87·8%) |
|  | High | 8,337 (24·2%) | 104 (5·1%) | 123 (4·9%) | 189 (11·7%) | 869 (22·7%) | 1,235 (28·4%) | 290 (19·9%) | 930 (32·1%) | 957 (29·6%) | 336 (28·8%) | 865 (32·5%) | 956 (23·8%) | 721 (29%) | 762 (34·8%) |
| **Primary safety outcome n (%)** | All | 5,126 (3·7%) | 386 (2·9%) | 483 (2·7%) | 320 (2·7%) | 406 (3·9%) | 425 (3·7%) | 131 (2·9%) | 311 (4·5%) | 347 (4·4%) | 110 (3·4%) | 640 (5·6%) | 681 (3·6%) | 690 (5·2%) | 196 (2·9%) |
|  | Low | 277 (0·5%) | 30 (0·4%) | 42 (0·4%) | 20 (0·3%) | <5 (*%) | <5 (*%) | <5 (*%) | 0 (0%) | 0 (0%) | 0 (0%) | 71 (1%) | 23 (0·2%) | 85 (1%) | <5 (*%) |
|  | Intermediate | 932 (2·2%) | 125 (3·9%) | 176 (4·1%) | 119 (3·8%) | <50 (*%) | <20 (*%) | <20 (*%) | 35 (1·1%) | 24 (0·6%) | 10 (0·7%) | 93 (4·6%) | 141 (2·5%) | 115 (5·8%) | <20 (*%) |
|  | High | 3,917 (11·4%) | 231 (11·4%) | 265 (10·5%) | 181 (11·2%) | 365 (9·5%) | 403 (9·3%) | 113 (7·8%) | 276 (9·5%) | 323 (10%) | 100 (8·6%) | 476 (17·9%) | 517 (12·9%) | 490 (19·7%) | 177 (8·1%) |
| **Index myocardial infarction n (%)** | All | 4,919 (3·6%) | 603 (4·5%) | 531 (3%) | 320 (2·7%) | 622 (6%) | 472 (4·2%) | 199 (4·5%) | 308 (4·5%) | 315 (4%) | 76 (2·4%) | 173 (1·5%) | 858 (4·5%) | 183 (1·4%) | 259 (3·8%) |
|  | Low | 74 (0·1%) | 7 (0·1%) | <5 (*%) | <5 (*%) | 0 (0%) | 0 (0%) | 0 (0%) | 0 (0%) | 0 (0%) | 0 (0%) | 14 (0·2%) | 43 (0·5%) | 6 (0·1%) | 0 (0%) |
|  | Intermediate | 179 (0·4%) | 30 (0·9%) | <25 (*%) | <20 (*%) | 0 (0%) | 10 (0·3%) | 0 (0%) | 0 (0%) | 0 (0%) | 0 (0%) | 9 (0·4%) | 85 (1·5%) | 6 (0·3%) | 0 (0%) |
|  | High | 4,666 (13·5%) | 566 (28%) | 507 (20·2%) | 301 (18·6%) | 622 (16·2%) | 462 (10·6%) | 199 (13·6%) | 308 (10·6%) | 315 (9·7%) | 76 (6·5%) | 150 (5·6%) | 730 (18·2%) | 171 (6·9%) | 259 (11·8%) |
| **Subsequent myocardial infarction n (%)** | All | 409 (0·4%) | 41 (0·3%) | 19 (0·1%) | 21 (0·2%) | 59 (0·6%) | 12 (0·1%) | 32 (0·7%) | 24 (0·4%) | 26 (0·3%) | 22 (0·7%) | # | 125 (0·7%) | # | 28 (0·4%) |
|  | Low | 18 (0·03%) | <5 (*%) | <5 (*%) | 0 (0%) | 0 (0%) | 0 (0%) | <5 (*%) | 0 (0%) | 0 (0%) | 0 (0%) | # | 10 (0·1%) | # | 0 (0%) |
|  | Intermediate | 87 (0·2%) | <10 (*%) | <5 (*%) | 6 (0·2%) | 10 (0·2%) | <5 (*%) | <15 (*%) | <5 (*%) | <5 (*%) | <5 (*%) | # | 30 (0·5%) | # | 7 (0·2%) |
|  | High | 304 (1%) | 32 (1·6%) | 11 (0·4%) | 15 (0·9%) | 49 (1·3%) | <15 (*%) | 19 (1·3%) | <25 (*%) | 23 (0·7%) | <20 (*%) | # | 85 (2·1%) | # | 21 (1%) |
| **All-cause mortality n (%)** | All | 3,985 (3·1%) | 348 (2·6%) | 465 (2·6%) | 302 (2·5%) | 350 (3·4%) | # | 102 (2·3%) | 290 (4·2%) | 324 (4·1%) | 90 (2·8%) | 464 (4%) | 558 (2·9%) | 524 (4%) | 168 (2·5%) |
|  | Low | 230 (0·4%) | 26 (0·3%) | 39 (0·4%) | 20 (0·3%) | <5 (*%) | # | 0 (0%) | 0 (0%) | 0 (0%) | 0 (0%) | 54 (0·8%) | <15 (*%) | 76 (0·9%) | <5 (*%) |
|  | Intermediate | 813 (2·1%) | 121 (3·8%) | 171 (4%) | 115 (3·7%) | <40 (*%) | # | 5 (0·2%) | 32 (1%) | 21 (0·6%) | 5 (0·4%) | 82 (4%) | <120 (*%) | 109 (5·5%) | <15 (*%) |
|  | High | 2,942 (9·8%) | 201 (9·9%) | 255 (10·1%) | 167 (10·3%) | 319 (8·3%) | # | 97 (6·7%) | 258 (8·9%) | 303 (9·4%) | 85 (7·3%) | 328 (12·3%) | 434 (10·8%) | 339 (13·6%) | 156 (7·1%) |
| **Any reattendance to the Emergency Department n (%)** | All | 19353 (14%) | 2312 (17·2%) | 2665 (15%) | 1867 (15·6%) | 1305 (12·7%) | 1065 (9·4%) | 555 (12·5%) | 2033 (29·7%) | 2233 (28·3%) | 452 (14·1%) | 769 (6·7%) | 1651 (8·7%) | 819 (6·2%) | 1627 (23·7%) |
|  | Low | 6504 (10·7%) | 1328 (16·2%) | 1478 (13·4%) | 969 (13·4%) | 175 (8·9%) | 233 (7%) | 88 (10·5%) | 190 (29·2%) | 339 (35·5%) | 65 (9·6%) | 300 (4·4%) | 702 (7·5%) | 418 (4·8%) | 219 (22·3%) |
|  | Intermediate | 6877 (16·1%) | 602 (18·8%) | 737 (17·1%) | 577 (18·4%) | 461 (10·3%) | 304 (8·3%) | 231 (10·7%) | 982 (29·8%) | 1109 (29·9%) | 149 (11%) | 187 (9·2%) | 539 (9·5%) | 166 (8·3%) | 833 (22·6%) |
|  | High | 5972 (17·3%) | 382 (18·9%) | 450 (17·9%) | 321 (19·8%) | 669 (17·5%) | 528 (12·2%) | 236 (16·2%) | 861 (29·7%) | 785 (24·3%) | 238 (20·4%) | 282 (10·6%) | 410 (10·2%) | 235 (9·4%) | 575 (26·2%) |

# Data unavailable

**Supplementary Table 2. Effectiveness and safety outcomes by hospital site for patients with possible myocardial infarction stratified by high-sensitivity cardiac troponin into low- (<5 ng/l), intermediate- (5 ng/L to sex-specific 99^th^ percentile), and high-risk (>sex-specific >99^th^ percentile) groups.**

| **Outcome** | **Risk stratification** | **All** | **Male** | **Female** | **Under 70 years of age** | **Over 70 years of age** | **White** | **Asian** | **Black** | **Other ethnicity** | **Deprivation group 1 (most deprived)** | **Deprivation group 2** | **Deprivation group 3 (least deprived)** |
| --- | --- | --- | --- | --- | --- | --- | --- | --- | --- | --- | --- | --- | --- |
| Primary effectiveness outcome n (%) | All | 72,961 (52·9%) | 36,838 (52·1%) | 36,113 (53·8%) | 58,533 (61·5%) | 14,428 (33·8%) | 35,138 (46·3%) | 10,026 (55·0%) | 5,458 (61·5%) | 8,603 (67·9%) | 12,090 (43%) | 22,830 (46·8%) | 6,697 (47·5%) |
|  | Low | 39,918 (65·8%) | 17,033 (66·1%) | 22,880 (65·5%) | 36,407 (67·5%) | 3,511 (51·6%) | 15,349 (59.3%%) | 4,572 (57·2%) | 2,659 (72·1%) | 6,167 (79·9%) | 7,998 (51·2%) | 12,877 (59·8%) | 3,193 (62·2%) |
|  | Intermediate | 24,706 (57·8%) | 15,763 (58·1%) | 8,938 (57·3%) | 18,556 (66·2%) | 6,150 (41·8%) | 14,579 (54·1%) | 4,301 (68·9%) | 2,041 (71·6%) | 1,727 (65·2%) | 3,132 (42·9%) | 7,739 (49·7%) | 2,606 (52·9%) |
|  | High | 8,337 (24·2%) | 4,042 (22·6%) | 4,295 (25·9%) | 3,570 (26·8%) | 4,767 (22·5%) | 5,210 (22·6%) | 1,153 (28·8%) | 758 (32·4%) | 709 (30·8%) | 960 (18·5%) | 2,214 (19%) | 898 (22·3%) |
| Primary safety outcome n (%) | All | 5,126 (3·7%) | 2,998 (4·2%) | 2,128 (3·2%) | 1,599 (1·7%) | 3,527 (8·3%) | 3,439 (4·5%) | 482 (2·6%) | 282 (3·2%) | 478 (3·8%) | 766 (2·7%) | 1,609 (3·3%) | 558 (4%) |
|  | Low | 277 (0·5%) | 152 (0·6%) | 125 (0·4%) | 178 (0·3%) | 99 (1·5%) | 136 (0·5%) | 21 (0·3%) | 23 (0·6%) | 50 (0·6%) | 38 (0·2%) | 65 (0·3%) | 17 (0·3%) |
|  | Intermediate | 932 (2·2%) | 660 (2·4%) | 272 (1·7%) | 340 (1·2%) | 592 (4%) | 650 (2·4%) | 88 (1·4%) | 37 (1·3%) | 75 (2·8%) | 174 (2·4%) | 352 (2·3%) | 119 (2·4%) |
|  | High | 3,917 (11·4%) | 2,186 (12·2%) | 1,731 (10·4%) | 1,081 (8·1%) | 2,836 (13·4%) | 2,653 (11·5%) | 373 (9·3%) | 222 (9·5%) | 353 (15·4%) | 554 (10·7%) | 1,192 (10·2%) | 422 (10·5%) |
| Index myocardial infarction n (%) | All | 4,919 (3·6%) | 3,282 (4·6%) | 1,637 (2·4%) | 2,701 (2·8%) | 2,218 (5·2%) | 3,231 (4·3%) | 709 (3·9%) | 158 (1·8%) | 237 (1·9%) | 897 (3·2%) | 2,056 (4·2%) | 716 (5·1%) |
|  | Low | 74 (0·1%) | 49 (0·2%) | 25 (0·1%) | 66 (0·1%) | 8 (0·1%) | 14 (0·1%) | <5 (*%) | <5 (*%) | 9 (0·1%) | <5 (*%) | 38 (0·2%) | 12 (0·2%) |
|  | Intermediate | 179 (0·4%) | 147 (0·5%) | 32 (0·2%) | 116 (0·4%) | 63 (0·4%) | 119 (0·4%) | 19 (0·3%) | <5 (*%) | <15 (*%) | 40 (0·5%) | 93 (0·6%) | 31 (0·6%) |
|  | High | 4,666 (13·5%) | 3,086 (17·3%) | 1,580 (9·5%) | 2,519 (18·9%) | 2,147 (10·2%) | 3,098 (13·5%) | 688 (17·2%) | 151 (6·5%) | 216 (9·4%) | 853 (16·4%) | 1,925 (16·5%) | 673 (16·7%) |
| Subsequent myocardial infarction n (%) | All | 409 (0·4%) | 275 (0·5%) | 134 (0·2%) | 225 (0·3%) | 184 (0·5%) | 284 (0·4%) | 61 (0·4%) | 9 (0·2%) | 8 (0·2%) | 82 (0·3%) | 176 (0·4%) | 72 (0·5%) |
|  | Low | 18 (0·03%) | 12 (0·1%) | 6 (0%) | <20 (*%) | <5 (*%) | 5 (0%) | <5 (*%) | 0 (0%) | 0 (0%) | <5 (*%) | 11 (0·1%) | <5 (*%) |
|  | Intermediate | 87 (0·2%) | 67 (0·3%) | 20 (0·1%) | <65 (*%) | <25 (*%) | 62 (0·2%) | <15 (*%) | <5 (*%) | <5 (*%) | <20 (*%) | 42 (0·3%) | <20 (*%) |
|  | High | 304 (1%) | 196 (1·3%) | 108 (0·8%) | 147 (1·3%) | 157 (0·9%) | 217 (1%) | 48 (1·4%) | <10 (*%) | <10 (*%) | 63 (1·2%) | 123 (1·1%) | 53 (1·3%) |
| All-cause mortality n (%) | All | 3985 (3·1%) | 2,273 (3·5%) | 1,712 (2·8%) | 1,093 (1·2%) | 2,892 (7·5%) | 2,682 (4·0%) | 384 (2·1%) | 222 (2·6%) | 338 (2·8%) | 520 (2·2%) | 1,265 (2·9%) | 424 (3·4%) |
|  | Low | 230 (0·4%) | 123 (0·5%) | 107 (0·3%) | 139 (0·3%) | 91 (1·4%) | 118 (0·5%) | 17 (0·2%) | 19 (0·5%) | 41 (0·5%) | 33 (0·2%) | 53 (0·3%) | 13 (0·3%) |
|  | Intermediate | 813 (2·1%) | 565 (2·3%) | 248 (1·7%) | 257 (1%) | 556 (4%) | 574 (2·4%) | 75 (1·2%) | 32 (1·2%) | 59 (2·4%) | 150 (2·5%) | 305 (2·2%) | 102 (2·3%) |
|  | High | 2942 (9·8%) | 1,585 (10·1%) | 1,357 (9·4%) | 697 (5·8%) | 2,245 (12·3%) | 1,990 (10·3%) | 292 (7·4%) | 171 (7·7%) | 238 (10·9%) | 337 (9·1%) | 907 (9·6%) | 309 (9·1%) |

**Supplementary Table 3. Effectiveness and safety outcomes by subgroup for patients with possible myocardial infarction stratified by high-sensitivity cardiac troponin into low- (<5 ng/l), intermediate- (5 ng/L to sex-specific 99^th^ percentile), and high-risk (>sex-specific >99^th^ percentile) groups.**

| **Management** | **All** | **Site A** | **Site B** | **Site C** | **Site D** | **Site E** | **Site F** | **Site G** | **Site H** | **Site I** | **Site J** | **Site K** | **Site L** | **Site M** |
| --- | --- | --- | --- | --- | --- | --- | --- | --- | --- | --- | --- | --- | --- | --- |
| High risk patients n | 34,447 | 2,024 | 2,515 | 1,619 | 3,831 | 4345 | 1,458 | 2,901 | 3,233 | 1,167 | 2,660 | 4,012 | 2,490 | 2,192 |
| Coronary angiography within 30 days n (%) | 1604 (6·1%) | <5 (*%) | 36 (1·4%) | <5(*%) | 649 (16·9%) | # | 168 (11·5%) | 133 (4·6%) | 195 (6%) | 74 (6·3%) | 96 (3·6%) | # | 103 (4·1%) | 146 (6·7%) |
| Coronary revascularisation within 30 days n (%) | 1,523 (5·8%) | 374 (18·5%) | 336 (13·4%) | 245 (15·1%) | 74 (1·9%) | # | 13 (0·9%) | 149 (5·1%) | 172 (5·3%) | 7 (0·6%) | 13 (0·5%) | # | 19 (0·8%) | 121 (5·5%) |
| Any new anti-platelet therapy n (%) | 3,839 (21·6%) | 372 (18·4%) | 451 (17·9%) | 218 (13·5%) | 631 (16·5%) | # | 206 (14·1%) | # | # | 133 (11·4%) | 917 (34·5%) | # | 911 (36·6%) | # |
| New dual anti-platelet therapy n (%) | 3,049 (17·2%) | 187 (9·2%) | 210 (8·3%) | 102 (6·3%) | 634 (16·5%) | # | 221 (15·2%) | # | # | 105 (9%) | 803 (30·2%) | # | 787 (31·6%) | # |
| New lipid lowering therapy n (%) | 4,059 (22·8%) | 409 (20·2%) | 514 (20·4%) | 240 (14·8%) | 491 (12·8%) | # | 155 (10·6%) | # | # | 96 (8·2%) | 1,098 (41·3%) | # | 1,056 (42·4%) | # |
| New beta blocker n (%) | 1,295 (7·3%) | 32 (1·6%) | 48 (1·9%) | 21 (1·3%) | 668 (17·4%) | # | 207 (14·2%) | # | # | 188 (16·1%) | 66 (2·5%) | # | 65 (2·6%) | # |
| New ACE inhibitor or ARB 2 antagonist n (%) | 3,615 (20·4%) | 318 (15·7%) | 337 (13·4%) | 176 (10·9%) | 438 (11·4%) | # | 146 (10%) | # | # | 101 (8·7%) | 1,063 (40%) | # | 1,036 (41·6%) | # |
| New outpatient appointment n (%) | 11,215 (43%) | 1,297 (64·1%) | 1,404 (55·8%) | 962 (59·4%) | 1,247 (32·6%) | # | 459 (31·5%) | 1,261 (43·5%) | 1,472 (45·5%) | 369 (31·6%) | 948 (35·6%) | # | 889 (35·7%) | 907 (41·4%) |

# Data unavailable

**Supplementary Table 4. Management outcomes by hospital site for patients with possible myocardial infarction who were stratified by high-sensitivity troponin as high-risk (>sex-specific >99^th^ percentile)**

| **Management** | **All** | **Male** | **Female** | **Under 70 years of age** | **Over 70 years of age** | **White** | **Asian** | **Black** | **Other ethnicity** | **Deprivation group 1 (most deprived)** | **Deprivation group 2** | **Deprivation group 3 (least deprived)** |
| --- | --- | --- | --- | --- | --- | --- | --- | --- | --- | --- | --- | --- |
| n | 34,447 | 17,877 | 16,568 | 13,298 | 21,149 | 23,027 | 4,004 | 2,338 | 2,299 | 5,191 | 11,656 | 4,024 |
| Coronary angiography  n (%) | 1,604 (6·1%) | 1,154 (8·4%) | 450  (3·6%) | 1,073 (10·3%) | 531  (3·4%) | 995  (6·3%) | 280  (7·3%) | 57  (2·6%) | 108  (5·1%) | 142  (4·5%) | 544  (7·8%) | 236  (9·7%) |
| Coronary revascularisation n (%) | 1,523 (5·8%) | 1,043 (7·6%) | 480  (3·9%) | 973  (9·4%) | 550  (3·5%) | 845  (5·3%) | 373  (9·7%) | 72  (3·3%) | 63  (3%) | 361 (11·5%) | 529  (7·6%) | 159  (6·5%) |
| Any new anti-platelet therapy n (%) | 3,839 (21·6%) | 2,165 (25·1%) | 1,674 (18·3%) | 1,591 (24·5%) | 2,248 (20%) | 2,260 (18·9%) | 430 (32·4%) | 313 (29·8%) | 563 (33·5%) | 559 (17·9%) | 1,075 (15·4%) | 365  (15%) |
| New dual anti-platelet therapy n (%) | 3,049 (17·2%) | 1,873 (21·8%) | 1,176 (12·8%) | 1,342 (20·6%) | 1,707 (15·2%) | 1,760 (14·7%) | 334 (25·2%) | 255 (24·3%) | 490 (29·1%) | 367 (11·7%) | 798 (11·4%) | 287 (11·8%) |
| New lipid lowering therapy n (%) | 4,059 (22·8%) | 2,200 (25·6%) | 1,859 (20·3%) | 1,510 (23·2%) | 2,549 (22·6%) | 2,324 (19·5%) | 481 (36·3%) | 351 (33·5%) | 644 (38·3%) | 579 (18·5%) | 1,006 (14·4%) | 310 (12·7%) |
| New beta blocker  n (%) | 1,295 (7·3%) | 744 (8·6%) | 551 (6%) | 655 (10·1%) | 640 (5·7%) | 981 (8·2%) | 53 (4%) | 37 (3·5%) | 60 (3·6%) | 191 (6·1%) | 665 (9·5%) | 296 (12·1%) |
| New ACE inhibitor or ARB 2 antagonist n (%) | 3,615 (20·4%) | 1,988 (23·1%) | 1,627 (17·8%) | 1,445 (22·2%) | 2,170 (19·3%) | 1,957 (16·4%) | 377 (28·4%) | 414 (39·5%) | 666 (39·6%) | 417 (13·3%) | 821 (11·8%) | 267 (11%) |
| New outpatient appointment n (%) | 11,215 (43%) | 6,157 (45%) | 5,058 (40·8%) | 4,992 (48%) | 6,223 (39·7%) | 6,701 (42·2%) | 1,858 (48·5%) | 987 (45·3%) | 823 (38·7%) | 1,643 (52·5%) | 3,067 (43·9%) | 1,006 (41·3%) |

**Supplementary Table 5. Management outcomes by subgroup for patients with possible myocardial infarction who were stratified by high-sensitivity troponin as high-risk (>sex-specific >99^th^ percentile)**

**Supplementary Table 6. Logistic regression modelling, within our nested study (n=17,934), demonstrating the relationship between risk groups and the primary effectiveness and safety outcomes adjusted for age, sex, ethnicity, and socioeconomic deprivation.**

|  | **Odds Ratio (95% CI)**  **Primary effectiveness outcome** | **Odds Ratio (95% CI)**  **Primary safety outcome** |
| --- | --- | --- |
| **Unadjusted model** | | |
| Group  Low risk  Intermediate risk  High risk | Reference  0·31 (0·27 to 0·34))  0·05 (0·04 to 0·05) | Reference  14·69 (4·61 to 89·50)  171·0 (55·16 to 1030·54) |
| **Adjusted for sex, age, ethnicity, and socioeconomic deprivation status** | | |
| Group  Low risk  Intermediate risk  High risk | Reference  0·39 (0·35 to 0·44)  0·06 (0·06 to 0·07) | Reference  9·65 (2·98 to 59·19)  88·08 (27·49 to 538·11) |

**Supplementary Table 7. Logistic regression modelling, within out nested study (n=17,934), demonstrating the relationship between subgroups and the primary effectiveness and safety outcomes adjusted for age, sex, ethnicity, and socioeconomic deprivation.**

|  | **Odds Ratio (95% CI)**  **Primary effectiveness outcome** | **Odds Ratio (95% CI)**  **Primary safety outcome** | |
| --- | --- | --- | --- |
| **Unadjusted model** | | |  |
| Sex  Female  Male  Age  <70 years of age  >70 years of age  Ethnicity  White  Asian  Black  Other ethnicity  Social deprivation  Group 1 (most deprived)  Group 2  Group 3 (least deprived) | Reference  0·77 (0·72 to 0·81)  Reference  0·26 (0·25 to 0·28)  Reference  2·07 (1·74 to 2·48)  2·02 (1·49 to 2·78)  1·70 (1·34 to 2·17)  1·02 (0·94 to 1·11)  Reference  1·00 (0·93 to 1·08) | Reference  1·51 (1·28 to 1·77)  Reference  4·68 (3·97 to 5·54)  Reference  0·21 (0·07 to 0·45)  0·13 (0·01 to 0·60)  0·26 (0·06 to 0·68)  1·19 (0·96 to 1·46)  Reference  1·23 (1·02 to 1·48) | |
| **Adjusted for sex, age, ethnicity and socioeconomic deprivation status** | | |  |
| Sex  Female  Male  Age  <70 years of age  >70 years of age  Ethnicity  White  Asian  Black  Other ethnicity  Social deprivation  Group 1 (most deprived)  Group 2  Group 3 (least deprived) | Reference  0·70 (0·66 to 0·74)  Reference  0·26 (0·24 to 0·27)  Reference  1·64 (1·37 to 1·97)  1·38 (1·01 to 1·92)  1·25 (0·98 to 1·62)  0·92 (0·84 to 1·00)  Reference  1·16 (1·07 to 1·25) | Reference  1·63 (1·39 to 1·93)  Reference  4·69 (3·96 to 5·57)  Reference  0·30 (0·11 to 0·65 )  0·24 (0·01 to 1·08)  0·41 (0·10 to 1·09)  1·39 (1·11 to 1·72)  Reference  1·05 (0·87 to 1·27) | |

**Supplementary Table 8. Presenting complaint leading to measurement of cardiac troponin across hospital sites D, F and I (n=17,934)**

| **Presenting complaint** | **All (n=17,934)** | **Site D (n=10,281)** | **Site F (n=4,452)** | **Site I**  **(n=3,201)** |
| --- | --- | --- | --- | --- |
| **Chest pain** | 11,874 (86·2%) | 6809 (86·0%) | 3016 (89·2%) | 2049 (82·8%) |
| **Palpitation** | 387 (2·8%) | 174 (2·2%) | 77 (2·3%) | 136 (5·5%) |
| **Dyspnoea** | 411 (3·0%) | 203 (2·6%) | 107 (3·2%) | 101 (4·1%) |
| **Syncope** | 308 (2·2%) | 192 (2·4%) | 77 (2·3%) | 39 (0·1%) |
| **Other** | 791 (5·7%) | 535 (6·7%) | 106 (3·1%) | 150 (6·1%) |
| **Data available** | 13,771 (76·8%) | 7913 (76·9%) | 3383 (76·0%) | 2475 (77·3%) |

**Supplementary Table 9. Primary effectiveness outcome for all patients presenting to hospital and for patients presenting to hospital with symptom recorded Sites D, F and I (n=13,771)**

|  | **Proportion of patients discharged with any symptom** |  | **Proportion of patients discharged with chest pain** |
| --- | --- | --- | --- |
| **All Sites (n=13,771)** | 9,850 (71·5%) | **All Sites (n=11,874)** | 6,771 (57·0%) |
| **Site D (n=7,913)** | 5,252 (66·4%) | **Site D (n=6,809)** | 3,535 (51·9%) |
| **Site F (n=3,383)** | 2,551 (75·4%) | **Site F (n=3,016)** | 1,827 (60·6%) |
| **Site I (n=2,475)** | 2,047 (82·7%) | **Site I (n=2,049)** | 1,409 (68·8%) |

**Supplementary Table 10. Primary safety outcome for all patients presenting to hospital and for patients presenting to hospital with symptom recorded at Sites 1, 2 and 3 (n=13,771)**

|  | **Proportion of patients with the primary safety outcome with any symptom** |  | **Proportion of patients with the primary safety outcome with chest pain** |
| --- | --- | --- | --- |
| **All Sites (n=13,771)** | 647 (4·7%) | **All Sites (n=11,874)** | 291 (2·5%) |
| **Site D (n=7,913)** | 406 (5·1%) | **Site 1 (n=6,809)** | 176 (2·6%) |
| **Site F (n=3,383)** | 131 (3·9%) | **Site 2 (n=3,016)** | 60 (2·0%) |
| **Site I (n=2,475)** | 110 (4·4%) | **Site 3 (n=2,049)** | 55 (2·7%) |

**
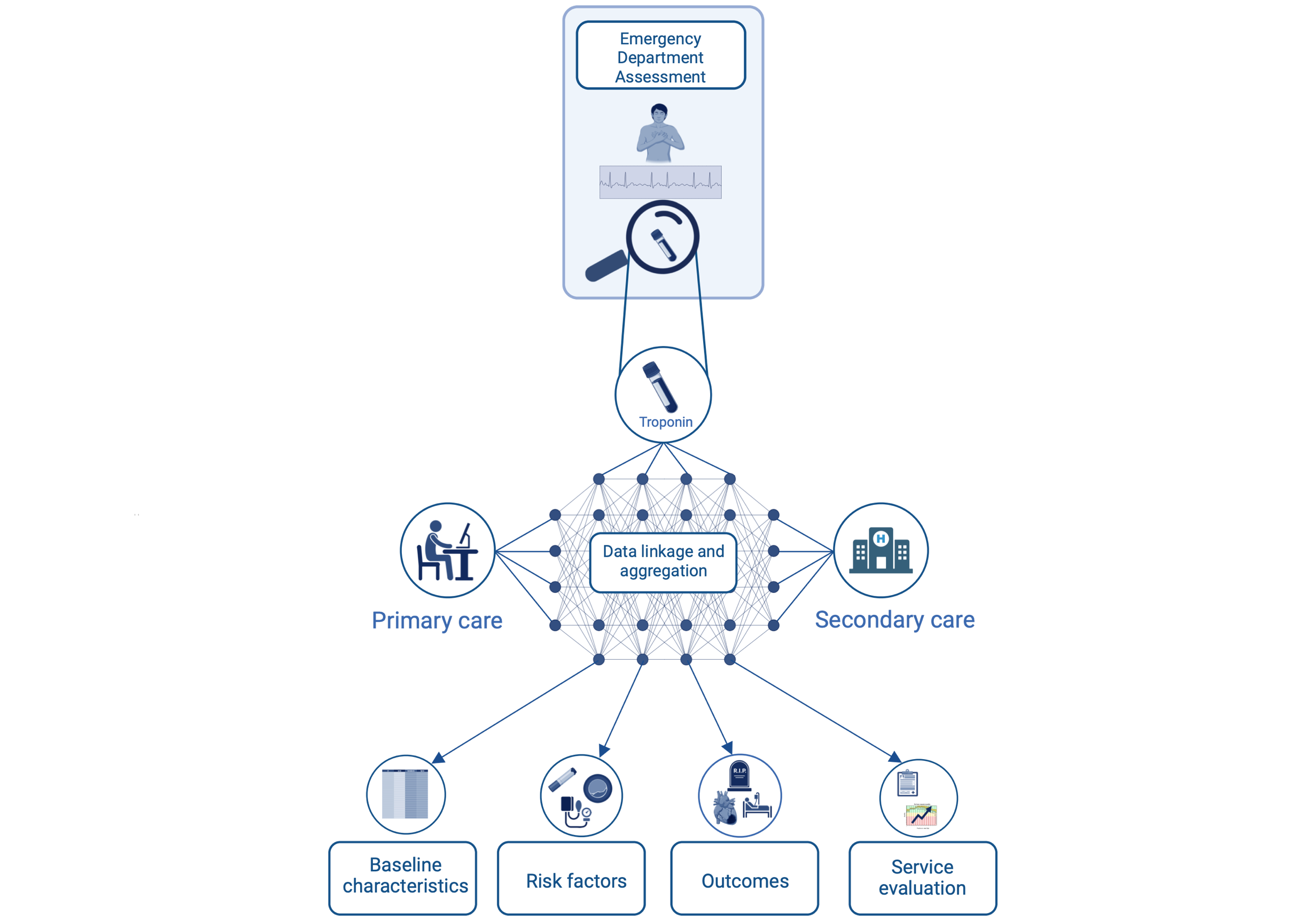
**

**Supplementary Figure 1. Overview of methodology using laboratory data (high sensitivity cardiac troponin) to identify patients who presented with possible myocardial infarction to allow characterisation of their demographics, risk factors and outcomes from their assessment in the Emergency Department.**

**Supplementary Figure 2. Negative predictive value of high-sensitivity cardiac troponin I or T concentrations <5 ng/L for the primary safety outcome (composite of all cause death or subsequent myocardial infarction at 30 days post hospital discharge) by hospital site, separated by high sensitivity cardiac troponin assay.**

**Supplementary Figure 3. Proportion of patients discharged from the emergency department stratified by high-sensitivity cardiac troponin concentration into low- (<5 ng/l), intermediate- (5 ng/L to sex-specific 99^th^ percentile), and high-risk (>sex-specific >99^th^ percentile) groups by hospital site and use of adjuvant risk score (HEART score) or not.**

**Supplementary Figure 4 Proportion of patients discharged from the emergency department stratified by high-sensitivity cardiac troponin concentration into low- (<5 ng/l), intermediate- (5 ng/L to sex-specific 99^th^ percentile), and high-risk (>sex-specific >99^th^ percentile) groups by hospital site and whether a site is a secondary or tertiary centre.**

**Supplementary Figure 5. Proportion of patients discharged from hospital (A) and admitted to hospital (B) stratified by high-sensitivity cardiac troponin concentration into low- (<5 ng/l), intermediate- (5 ng/L to sex-specific 99^th^ percentile), and high-risk (>sex-specific >99^th^ percentile) groups by hospital site and cardiac troponin assay.**

**Supplementary Figure 6. Proportion of patients discharged from hospital stratified by high-sensitivity cardiac troponin concentration into low- (<5 ng/l), intermediate- (5 ng/L to sex-specific 99^th^ percentile), and high-risk (>sex-specific >99^th^ percentile) groups by hospital site with any symptom being the presenting complaint (A) and with chest pain (B).**

**Supplementary Figure 7. Negative predictive value of high-sensitivity cardiac troponin I or T concentrations <5 ng/L for the primary safety outcome (composite of all cause death or subsequent myocardial infarction at 30 days post hospital discharge) for patients presenting with any symptom (n=13,771)**

**Supplementary Figure 8. Negative predictive value of high-sensitivity cardiac troponin I or T concentrations <5 ng/L for the primary safety outcome (composite of all cause death or subsequent myocardial infarction at 30 days post hospital discharge) for patients presenting with chest pain (n=11,874)**
